# Supplementary figures and images for: Mechanistic modeling of cell viability assays with in silico lineage tracing
Source: PLoS Comput Biol. 2025 Aug 29;21(8):e1013156. doi: 10.1371/journal.pcbi.1013156 (PMC12416836; doi:10.1371/journal.pcbi.1013156)

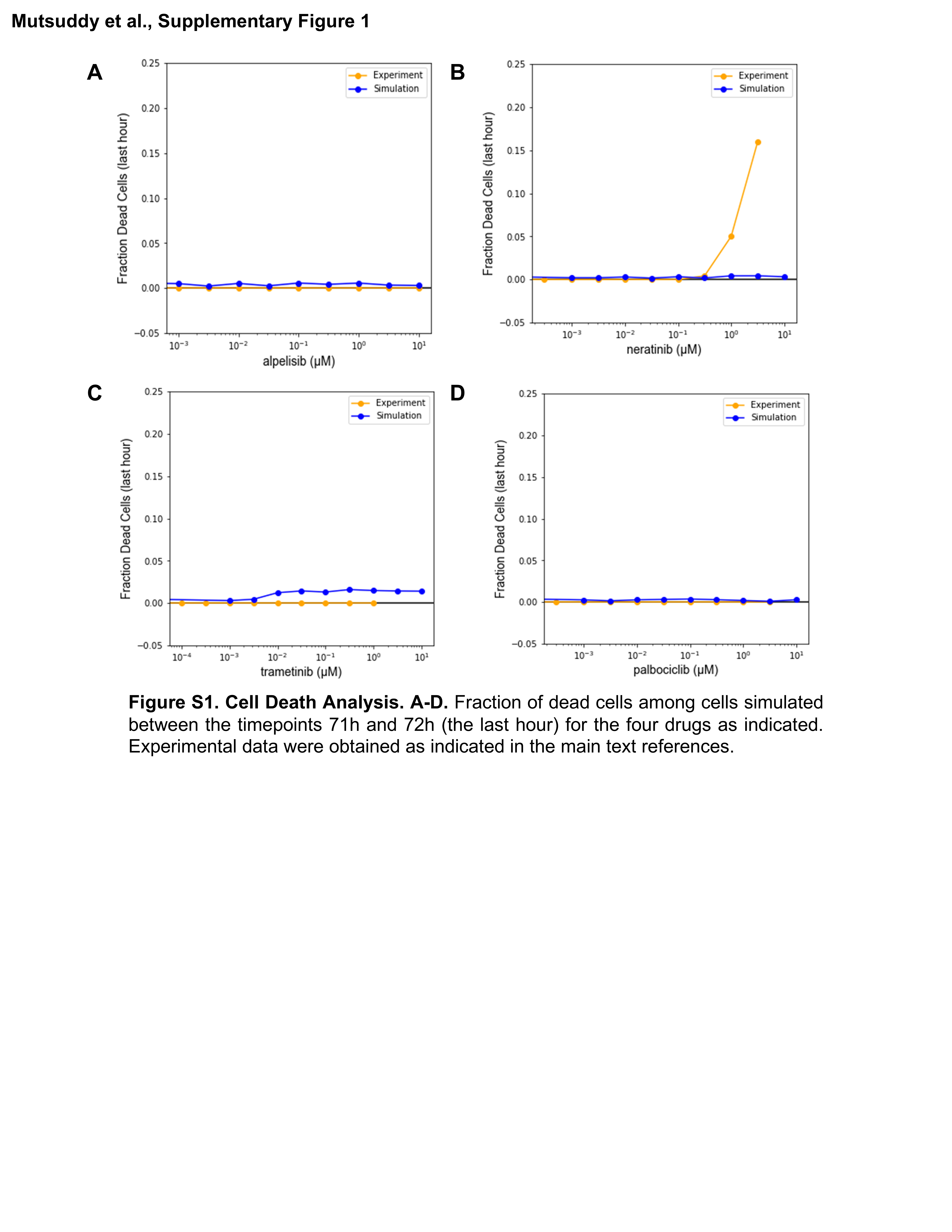

Supplement: S1 Fig — A-D. Fraction of dead cells among cells simulated between the timepoints 71h and 72h (the last hour) for the four drugs as indicated. Experimental data were obtained as indicated in the main text references. (TIF) [file pcbi.1013156.s001.tif]

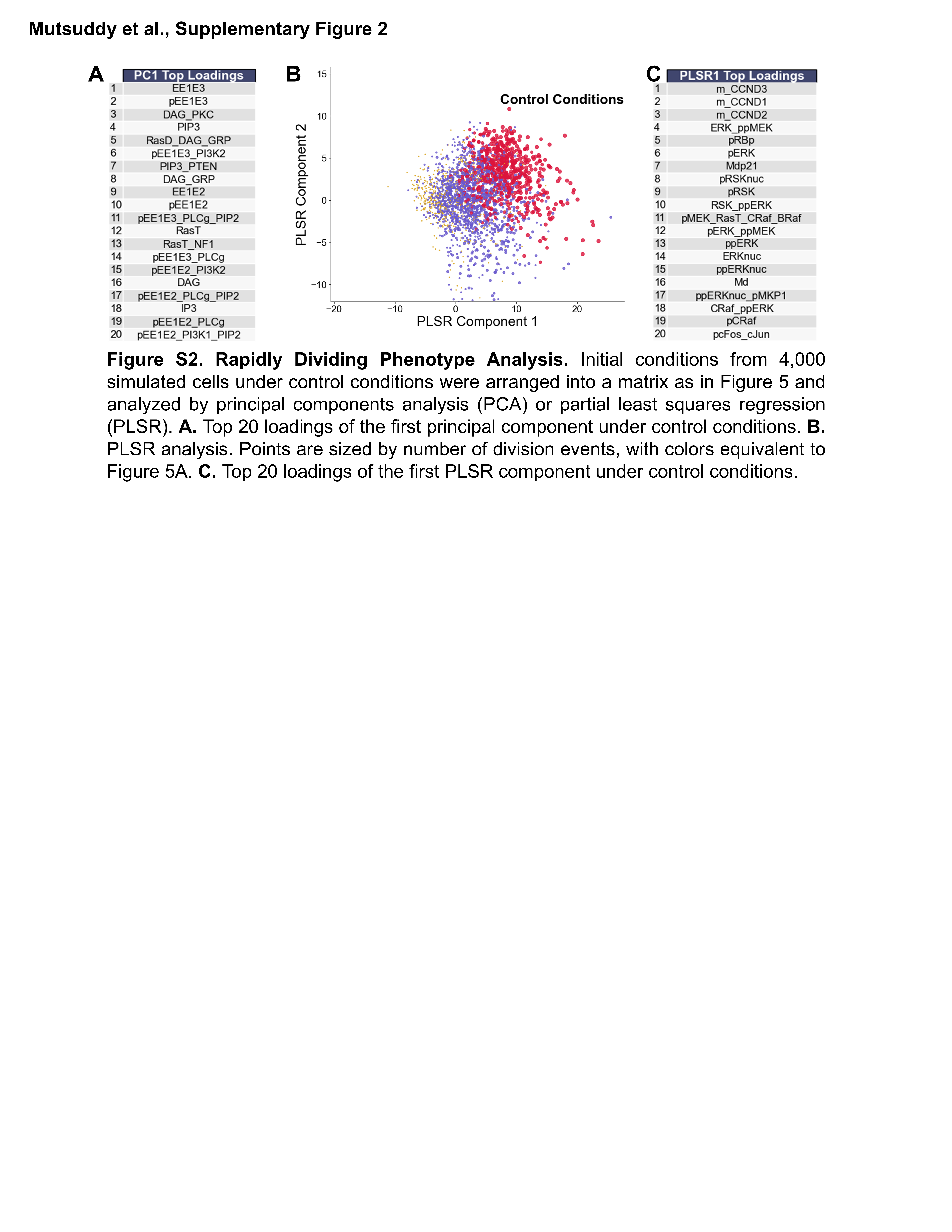

Supplement: S2 Fig — Initial conditions from 4,000 simulated cells under control conditions were arranged into a matrix as in Fig 5 and analyzed by principal components analysis (PCA) or partial least squares regression (PLSR). A. Top 20 loadings of the first principal component under control conditions. B. PLSR analysis. Points are sized by number of division events, with colors equivalent to Fig 5A. C. Top 20 loadings of the first PLSR component under control conditions. (TIF) [file pcbi.1013156.s002.tif]
